# Considerations on the late-preterm newborn with cardiac problems

**DOI:** 10.1186/1824-7288-40-S2-A50

**Published:** 2014-10-09

**Authors:** S Fiocchi

**Affiliations:** 1 Terapia Intensiva Neonatale, Ospedale Niguarda Ca’ Granda, Milano, Italy

## 

Focus on the late preterm birth (LPB) and critical congenital heart defects (CCHD)

Data on outcomes of CCHD specifically in late preterm infants are exceedingly scarce.

The preterm newborn with associated complex CHD presents many intricate problems to clinicians who take care (neonatologist, cardiologist, cardiothoracic surgeon). Therapies used for the preterm neonate without heart disease frequently need to be altered in the presence of heart disease and those needed for management of heart disease may need to be altered because of prematurity.

The population of newborn born preterm is at higher risk for postoperative death and short and long term complications than term newborn with the same CHD and the causes of this are multifactorial. Technical issue related to small cardiac structure, immaturity of other organ systems, decreased nutritional and cardiac reserve, increased risks of bleeding, abnormal chest wall mechanics after sternotomy can all together account for this epidemiologic data. In past years these findings were related in particular to low-birth weight infants and conventional management was oriented to await a threshold weight in order to reduce bypass-related morbidities. From 1990 rates of LPB have risen and now account for 75% of all preterm births. Whether late preterm infants with CHD, who may be only marginally more immature than their term counterparts, remain at risk for adverse outcome in unclear. Some recent papers do demonstrate the independent effect of LPB on mortality and morbidity if a CHD is present and this issue emerge as an important factor for a correct counseling. Natarajan have observed that the weight at surgical intervention was significantly lower and age higher in the late preterm infants compared to those delivered at term. Late preterm infants had significantly higher rates of NEC and seizures, with a greater risk of supplemental oxygen and tube feeding at discharge. Costello have examined mortality and morbidity stratified by gestational age, reporting for LPB group with CHD an hospital mortality rate of 16.4% compared to 2.6-8% in the term group. The exact mechanism of this vulnerability remains unclear although functional immaturity of the lung and the brain are plausible aetiologies. Altered intrinsic cardiac mechanism could play a role in adverse outcome of late preterm with CHD. Gestational age at delivery can be a risk factor over which clinicians might have some control. Before birth the challenge is planning the time and the place for the delivery of a foetus with CHD. After birth the challenge is not waiting for obtain an arbitrary correct weight for surgery but working in a complex team to discuss each case in order to maximize outcome.

Domande inerenti la relazione del Dott. Stefano Fiocchi

## Considerations in the late-preterm newborn with cardiac problems

Una cardiopatia congenita critica è:

a) Quasi sempre mortale

b) se esiste una ostruzione “critica” agli efflussi ventricolari

c) se richiede l’uso delle prostaglandine

d) **se richiede intervento chirurgico nel primo mese di vita**

Qual’è il peso ottimale per la correzione chirurgica di una cardiopatia congenita critica?

a) **Non esiste un peso “ottimale”**

b) > 2000 gr

c) > 2500 gr

d) > 3000 gr

Il rischio di mortalità neonatale nel late preterm affetto da cardiopatia congenita critica è:

a) 8%

b) < 5%

c) 25-30%

d) **15-20%**

La aumentata vulnerabilità del late preterm con cardiopatia congenita si associa a:

a) Ventilazione prolungata

b) Prolungato uso delle prostaglandine

c) Presenza di altre malformazioni associate

d) **Tutte le precedenti**

